# The Effect of a Training Program Based on the PRECEDE-PROCEED Model on Lifestyle of Adolescents with Beta-Thalassemia: A Randomized Controlled Clinical Trial

**Published:** 2019-01-01

**Authors:** Malihe Bazpour, Mahin Gheibizadeh, Amal Saki Malehi, Bijan Keikhaei

**Affiliations:** 1 Department of Nursing, School of Nursing and Midwifery, Ahvaz Jundishapur University of Medical Sciences, Ahvaz, Iran; 2 Nursing Care Research in Chronic Disease, Ahvaz Jundishapur University of Medical Sciences, Ahvaz, Iran; 3 Department of Biostatistics and Epidemiology, School of Public Health, Ahvaz Jundishapur University of Medical Sciences, Ahvaz, Iran; 4 Department of Pediatric Hematology and Oncology, Ahvaz Jundishapur University of Medical Sciences, Ahvaz, Iran

**Keywords:** Beta-thalassemia, Lifestyle, PRECEDE-PROCEED model, Education

## Abstract

**Background:** Lifestyle is a key issue in the concept of health promotion. Lifestyle includes all activities that encourage optimum physical, spiritual, and mental functions. The aim of this study was to determine the effect of a training program based on PRECEDE-PROCEED Model on lifestyle of adolescents with beta thalassemia.

**Materials and Methods:** In this clinical trial study, 64 adolescents (age 16-20) who referred to the Thalassemia Center of Ahvaz (2015) were selected and randomly divided into two groups: experimental and control group. The components of the PRECEDE-PROCEED Model were used for planning, implementation and evaluation of the program. Changes in predisposing, reinforcing, enabling factors and lifestyle were immediately and a month after the intervention were assessed by a questionnaire based on PRECEDE-PROCEED Model and the Health-Promoting Lifestyle Profile.

**Results:** The intervention had significantly positive effect on predisposing, enabling and reinforcing factors immediately and a month after the intervention (P < 0.05). Repeated measures analysis of variance showed a significant positive increase in the six dimensions of lifestyle score in the experimental group from baseline to one-month follow-up (P < 0.05).

**Conclusion:** This study showed that the theory-based training program in adolescents suffered from beta thalassemia disease could improve the adolescent’s awareness and attitude of healthy lifestyle.

## Introduction

 Beta-thalassemia is the most common inherited single gene disorder in the world^1^. Iran is one of the major centers for individuals with Beta-thalassemia among the Eastern Mediterranean Region. Considering the high consanguinity among the Iranian population, it is estimated that there are between two and three million Beta-thalassemia carriers and 25,000 patients in Iran^2^.

Nowadays, new treatment modalities have increased the longevity in patients with thalassemia major^2^. Therefore, patients with Beta-thalassemia have to deal with and manage their disease properly to enjoy a good life. On the other hand, Beta-thalassemia, similar to any other chronic disease, affects various aspects of a person’s life and people with the disease face serious problems in their health. Beta-thalassemia causes physical, emotional and social disorders such as osteoporosis, cardiac dysfunction, diabetes, and depression^3^^-^^8^. 

Nowadays, lifestyle strategies are receiving increased attention^9^ as it plays a significant role in bio-psychological health^10^. In fact, healthy lifestyle has been considered as a valuable source for decreasing health problems, promoting health, managing stressful events and improving the quality of life. This practice should be started in childhood and adolescence^11^. In Iran, 85% of patients with thalassemia are adolescents^12^, which increases the sensitivity to the health status of these affected patients^13^. Adolescence is the golden age to form a healthy lifestyle. World Health Organization (WHO) also believes that the fight against unhealthy way of life at an early age has a significant impact on health in adulthood ^14^.

Despite the importance of paying attention to lifestyle during adolescence, various studies conducted in Iran indicate that lifestyle in adolescents is undesirable^15^^-^^16^. It is believed that health education may result in lifestyle modifications. In general, health education may improve patients’ knowledge on a disease and its therapy which lead to better treatment adherence and taking a more positive role in the management of their health^17^. Education plays a central role in the management of chronic disease. Its main focus is to improve the behavior of individuals, groups and societies. Health education patterns and theories are used as an organized and basic tool for designing and evaluating education and health program interventions, as well as affecting influential forces on behavior and identifying the most appropriate target group^18^^-^^19^.

The PRECEDE-PROCEED Model was developed by Greene and Kreuter in 2002^20^.The efficiency of the PRECEDE-PROCEED Model has been proven in different studies in health^21^^-^^23^. There have been very few studies, with randomized controls, on the effects of education in lifestyle of patients with Beta-thalassemia. Therefore, the aim of this study was to assess the effectiveness of the PRECEDE-PROCEED education model on lifestyle of adolescents with Beta-thalassemia.

## Materials and Methods

**Study deign and sampling**

This randomized clinical trial study (IRCT2016011125954N3) was designed to assess the effect of a training program based PRECEDE-PROCEED Model on adolescents’ lifestyle referred to the Thalassemia Center between December 2015 and August 2016 in Ahvaz. According to the inclusion criteria, 64 adolescents were eligible to participate in the study. They were randomly divided into two groups: control and experimental group (32 in each group). Inclusion criteria for patients were: diagnosis of Beta-thalassemia, age 16 - 20 years, person's desire for participation, resident in Ahvaz, and having medical records in Thalassemia Center of Ahvaz. The exclusion criterion was having no -participation in one of the training sessions. 

**Data collection**

Data collected using two self-reported questionnaires; the Persian version of the Health-Promoting Lifestyle Profile II and a researcher-made questionnaire based on the PRECEDE-PROCEED Model. 

The Health-Promoting Lifestyle Profile (HPLP II) was first designed by Walker et al. in 1978^24^. In the current study, the Persian version of HPLP II created in 2012 by Zeidi was used^25^. This multidimensional tool assesses health-promoting behaviors in six aspects of nutrition (7 items), physical activity (7 items), responsibility for health (13 items), stress management (6 items), interpersonal relationship (8 items), and self-actualization (11 items). The questionnaire included 52 questions based on a four-item Likert scale: never =1, sometimes =2, often =3, and always = 4. The total score of health-promoting behaviors is between 52 and 208 with higher scores indicating a healthier lifestyle. For each aspect of behaviors, a separate score was calculated; therefore, the score of each subscale was calculated by the scores of the answers given to the questions of the same subscale. In each subscale and in total scale, achieving less than %50 of the total score indicates poor status, 50% to 75% represents an average status, and greater than 75% indicates a good status.

The researcher-made questionnaire based on PRECEDE-PROCEED Model was prepared based on information found in the literature review. The questionnaire had four sections as demographic information (including age, gender, education of the subject and his/her parents, and the occupation of his/her parents), predisposing factors (including 10 questions regarding knowledge and 15 questions regarding attitudes toward healthy lifestyle), enabling factors (including four questions to measure skills of stress management, interpersonal relations, self-actualization, access to and the use of training resources), and reinforcement factors (consisting of three questions to measure support and encouragement of peers, family and healthcare staff). Questions pertaining to knowledge, enabling factors, and reinforcement factors were scored in the form of Yes = 1 and No = 0, while the attitude questions were scored based on a 4-item Likert scale ranged from strongly disagree = 1 to strongly agree = 4. Therefore, the total score rang to predispose factors' section was 25-70, enabling factors' section 0-4, and reinforcement factors' section 0-3 (higher scores indicate better health status).

**Validity and reliability**

Content validity of the researcher-made questionnaire was determined using content validity index (CVI) and content validity rate (CVR). It was approved by 10 faculty members of Ahvaz Jundishapur University of Medical Sciences.  For the researcher-made questionnaire, CVR was 0.92 and CVI was 0.96. The reliability of the questionnaire was calculated using a test-retest method with two weeks’ interval. Test-retest was conducted on a sample of 15 individuals and Pearson correlation coefficient was calculated. Pearson correlation coefficient for all questions, knowledge, attitude, enabling factors, and reinforcing factors were 0.92, 0.89, 0.92, 0.90 and 0.89, respectively. 

The reliability of original version of HPLP II was determined by Walker, Sechrist and Pender (1978). Cronbach’s alpha for entire questionnaire and sections of self-actualization, health responsibility, physical activity, interpersonal relations, stress management, and nutrition were reported 0.94, 0.86, 0.86, 0.85, 0.87, 0.79 and 0.80, respectively^24^.

Validity and reliability of the Persian version of HPLP II was obtained by Zeidi et al. They reported Cronbach’s alpha of 0.64, 0.86, 0.75, 0.91, 0.79, 0.81, and 0.82 for self-actualization, health responsibility, interpersonal relations, stress management, physical activity, nutrition and the whole questionnaire, respectively ^25^.

**Data gathering process and the intervention**

The aim of the educational intervention was to obtain positive changes of lifestyle modifications in the six aspects of the HPLP in the experimental group. At first, the PRECEDE four- phase assessments were conducted and then an appropriate intervention based on the assessments was developed, implemented and evaluated.

**Social assessment**

In this phase, the researchers identified factors affecting lifestyle in target population. We used focus group discussions for data collection. Results showed that food habits, health behaviors, family, stress management can affect adolescent’s lifestyle. 

**Epidemiological, Behavioral, and Environmental assessment**

In this phase, we collected existing data regarding factors associated with lifestyle in Iran and other countries, using various online databases. Then, in behavioral and environmental assessments, factors causally associated with lifestyle were systematically identified, and the most important and changeable behavioral and environmental factors associated with lifestyle were found. Results of the focus group discussions were widely applied for this step. The most important factor found in adolescents was the lack of dominance of factors affecting lifestyle. In terms of environmental determinants of the healthy lifestyle, access to place, people, or informational resources such as educational materials, classes and databases were considered as the target behavior. Some demographic variables such as parents’ occupation and education were also considered as non-health factors related to lifestyle in target population. 

**Educational and ecological assessments**

This phase entails identifying the predisposing, enabling, and reinforcing factors, which leads to behavioral change. Predisposing factors are antecedents to behaviors that motivate particular health-related behaviors such as knowledge and attitude related to lifestyle. Reinforcing factors are provided in reward and incentive for the persistence of the health-related behavior such as getting influence from significant people. In this study, getting support and encouragement from family, peers, and health care workers considered as the reinforcing factors. Enabling factors are those that facilitate performance of the health behavior such as resources, skills, and supportive policies^26^. In this study, the enabling factors were availability and accessibility to educational resources and behavioral skills.

**Administrative and policy assessments**

The fourth phase of the model focuses on identifying resources, policies, supports, and facilities needed for implementing and evaluating the health education program^27^. We assessed a place and timetable for activities, budgeting, personnel, organizational barriers, facilitators, responsibilities, necessary supports and coordination for implementing educational interventions. After this assessment, the program's components were determined. Educational objectives, content of the educational program, messages, and materials were developed through finding expert's views and reviewing the scientific resources. Now, it was time to implement the program in the intervention group.

**Implementation**

After planning the training program, it was implemented among adolescents of the intervention group using lecture and group discussion. The training sessions were as follows:

1. The first session focused on healthy nutrition
2. The second session focused on physical activity
3. The third session focused on health responsibility
4. The fourth session focused on stress management
5. The fifth session focused on interpersonal relations
6. In the sixth session focused on spiritual growth.

Each session lasted 45 to 60 minutes. The researcher asked the participants to transfer the training program to their families and peers. At the end of each session, the researcher gave them an instructional package containing necessary information related to lifestyle modification (about diet, stress management, physical activity, self-care, interpersonal relationship skills and self-actualization).

**Process evaluation**

Process evaluation occurs during implementation of the program and is used to evaluate the process by which the program is being operated. In this phase, achieving the educational objectives is measured^28^. In this study, process evaluation includes evaluating the program components such as the program staff, methods, materials used, and activities.

**Impact evaluation**

This phase determines the immediate effect of the program on the target behavior, and it occurs after the program ends^26^. In this study, impact evaluation consists of assessing changes in predisposing, reinforcing, and enabling factors which affects the behavior immediately after and one month after intervention activities through analysis of the questionnaires.

**Data analysis**

The data were analyzed using SPSS 22 statistical software as well as descriptive and inferential statistics. Descriptive analysis carried out for each variable included in the study: mean, standard deviation and frequencies with confidence intervals of 95% (95% CI) for quantitative variables and qualitative variables, respectively. 

Mann-Whitney U test was used to compare the two groups of experimental and control. ANOVA with repeated measures was used to determine the adjusted effect of educational program based on the PRECEDE-PROCEED model. In all instances, the accepted level of significance was 0/05 or less, with 95% CI. 

**Ethical considerations**

The Ethics Committee of Ahvaz Jundishapour University of Medical Sciences (IR.AJUMS.REC.1394.507) approved the study. In the study, the researchers were committed to the ethical issues such as voluntary participation in research, obtaining consent for participation in the project and informing the participants of the purpose of the study.

## Results

 Demographic characteristics at baseline are summarized in Tables 1 and 2. No significant differences were found between the two groups in terms of the demographic measures. Before intervention, there was no significant difference between the two groups regarding the mean of knowledge, attitude, enabling factors, reinforcing factors, and lifestyle scores.

**Table 1:** Baseline characteristics of patient mean.

**Variable**		**Group**		**P-** **value**
		**Experimental. ** **N (%)**	**Control. ** **N (%)**	
Gender	Female	16 (50)	17 (53.1)	0.8
	Male	16 (50)	15(46.9)	
Father’s education level	Uneducated	1 (3.1)	3 (9.4)	0.48
	Under high School Diploma	13 (40.6)	8 (25)	
	Diploma	15 (46.9)	18 (56.2)	
	Master	3 (9.4)	3 (9.4)	
Mather’s education level	Uneducated	6 (18.8)	10 (31.2)	0.44
	Under high School Diploma	9 (28.1)	6 (18.8)	
	Diploma	17 (53.1)	16 (50)	
	Master	-	-	
Father’s occupation	Employee	8 (25)	7 (21.9)	0.21
	Worker	2 (6.3)	8 (25)	
	Pensioner	9 (28.1)	6 (18.8)	
	Self-employed	13 (40.6)	11 (34.4)	
Mather’s occupation	Housewife	3 (9.3)	2 (2.6)	0.64
	Employed	29 (90.7)	30 (97.4)	
Adolescent’s education level	Under high School Diploma	14 (43.8)	17 (53.1)	0.45
	Diploma	18 (56.2)	15 (46.9)	

**Table 2:** Qualitative characteristics of patient mean.

**Variable**	**Group**		**P-value**
	**Experimental**	**Control**	
Age	18.3 ± 1.37	17.84 ± 1.34	0.575
Thalassemia patient(s) in the family	1.31 ± 0.47	1.34 ± 0.48	0.792

Before intervention, the mean of knowledge score was in the weak level in the two groups, but immediately after and a month after intervention it reached to the good level in the experimental group, but no change was found in the control group. In terms of the mean of attitude score, there were no differences over time for the control group, but it reached to the moderate level immediately after and a month after intervention in the experimental group. The mean scores of enabling and reinforcing factors were also in weak level in both groups at baseline, but immediately after and a month after intervention they reached from moderate level to high level in the experimental group, while they remained in the weak level in the control group (Table 3).

**Table 3:** The mean and standard deviation of lifestyle and behavior analysis based on Precede-Proceed pre& post intervention

**Variables**	**Group**	**Before intervention**	**Immediately after ** **intervention**	**One month after ** **intervention**	**P-value**
Knowledge	Experimental	5.25 ± 1.13	9.87 ± 0.33	9.62 ± 0.48	> 0.001
	Control	5.09 ± 0.99	5.16 ± 1.05	5.34 ± 1.02	0.096
Attitude	Experimental	37 ± 4.59	57.09 ± 1.98	56.02 ± 2.01	> 0.001
	Control	36.15 ± 2.89	37.06 ± 2.81	36.34 ± 2.91	0.314
Enabling factors	Experimental	1.05 ± 0.62	3.84 ± 0.39	3.75 ± 0.43	> 0.001
	Control	0.94 ± 0.87	1.00 ± 0.87	1.04 ± 0.88	0.148
Reinforcing factors	Experimental	2.21 ± 0.55	3 ± 0.00	2.93 ± 0.24	> 0.001
	Control	1.96 ± 0.78	2.12 ± 0.60	2.06 ± 0.71	0.191
Lifestyle	Experimental	107.71 ± 7.93	186.48 ± 4.34	184.92 ± 4.01	> 0.001
	Control	104.31 ± 8.56	105.46 ± 7.78	106.09 ± 7.49	0.011

In this study, the means and standard deviations for the lifestyle were calculated at baseline, immediately and a month after intervention, in both the groups. The findings showed that the mean of the lifestyle score changed from 107.71 (weak level to moderate level) at baseline to 186.48 (good level) immediately after intervention, and finally reached 184.92 (good level) a month after intervention in the experimental group. But, there was no significant change in the mean of lifestyle score over time in the control group. 

As shown in Table 3, the mean of lifestyle score immediately and a month after intervention in the experimental group was higher than the control group (P < 0.001). In addition, the mean of lifestyle score immediately after and a month after the intervention was higher in the experimental group (P < 0.001). There was a significant correlation (P < 0.001) between the type of group and the mean of lifestyle score. In other words, changes in the mean of lifestyle score depend on which group belongs to the adolescents. 

The trend of changes in the behavior score over time was significant, so that this trend was very flat in the control group but had abrupt increase in the experimental group. Then, the significant effect of the intervention on the mean of lifestyle score was adjusted for confounding factors.

## Discussion

 The purpose of this study was to consider the effect of the training program based on the PRECEDE- PROCEED Model on the lifestyle of adolescents with beta-thalassemia. Our finding indicated that the PRECEDE- PROCEED Model is an appropriate model for planning and implementing the training program to promote healthier lifestyle. In this study, results revealed significantly positive predisposing (awareness and attitude), reinforcing, and enabling factor changes in the experimental group immediately after and a month after intervention. In contrast, no changes were observed in the control group. Furthermore, the overall score and the scores for 6 dimensions of the lifestyle (self-actualization, health responsibility, physical activity, nutrition, interpersonal relationship, and stress management) were increased in the post-test taken from the experimental group. Unfortunately, by searching the literature, there was no study on the effect of an educational program based on this model on the lifestyle of adolescents with β-thalassemia. Hence, the results were compared with those of other studies conducted in other populations. For example, our results were in line with previously reported findings of studies conducted by Soleiman Ekhtiari et al. on high school girls. They showed that after the intervention based on PRECEDE- PROCEED Model, the mean score of predisposing, reinforcing, and enabling factors in the experimental group significantly changed compared to the control group^29^. Our findings are in agreement with Solhi^30^, Ranjbaran^31^, Sabzmakan^32^ and Dizaji^33^ who reported that implementing interventions using PRECEDE Model could increase enabling and reinforcing factors. In Safabakhsh’s study, findings showed that Health Promoting Programs (HPP) improved patient’s lifestyle after coronary artery bypass^10^. In other studies, compared to baseline, lifestyle in the experimental group improved significantly after educational intervention. (Aghamolaei and Mehdi pour)^34^^-^^35^.

## CONCLUSION

 Overall, the results showed that the implementation of the training program based on the PRECEDE- PROCEED Model can lead to the promotion of lifestyle in adolescents with Beta- thalassemia. The PRECEDE-PROCEED Model can be applied as a conceptual framework for identifying the relevant behavioral and environmental factors associated with lifestyle.

In spite of the intervention's positive results on adolescents with Beta-thalassemia, this study had several limitations that deserve further discussions. This study lacked follow-up data to determine the long-term effects of the program on the quality of life and health status of target population. Further research needs to be carried out to examine the long-term effects of the program on health and quality of life indicators among adolescents with Beta-thalassemia. In addition, our study was based on self-reported information, which could be biased by the participants. We believe that the effectiveness of this intervention can be attributed to the use of the PRECEDE-PROCEED Model as a conceptual framework, which can play a significant role in improving the quality of training programs. In addition, we can implement and evaluate the health education programs through defined stages of this model.
